# Recommendations of the Spanish brachytherapy group of the Spanish Society of Radiation Oncology and the Spanish Society of Medical Physics for interstitial high-dose-rate brachytherapy for gynaecologic malignancies

**DOI:** 10.1007/s12094-022-03016-1

**Published:** 2022-11-29

**Authors:** Cristina Gutiérrez Miguélez, Silvia Rodríguez Villalba, Elena Villafranca Iturre, Naiara Fuentemilla Urio, Jose Richart Sancho, Sofía Córdoba Lago, Francisco Pino Sorroche, Ruth Gracia Lucio, Antonio Herreros Martínez, Dina Najjari-Jamal, GEB (Spanish Brachytherapy Group)

**Affiliations:** 1 Radiation Oncology Department, Institut Català d’Oncologia, Facultat de Medicina i Ciències de la Salut, Universitat de Barcelona (UB) Catalonia, Hospitalet de Llobregat, Spain; 2 Radiation Oncology Department, Hospital Clínica Benidorm, Benidorm, Spain; 3 Radiation Oncology Department, Complejo Hospitalario de Navarra, Pamplona, Spain; 4 Radiation Oncology Department, Hospital Universitario San Juan, Alicante, Spain; 5 Radiation Oncology Department, Hospital Universitario Puerta de Hierro, Madrid, Spain; 6 Radiophysics Department, Institut Català d’Oncologia, Hospitalet de Llobregat, Catalonia Spain; 7 Radiophysics Department, Hospital Clínic de Barcelona, Barcelona, Catalonia Spain

**Keywords:** Interstitial high-dose-rate gynaecological brachytherapy, Technical, dosimetric aspects, Recommendations, Consensus

## Abstract

The present document includes consensus-based recommendations from the Brachytherapy Group (GEB) of the Spanish Society of Radiation Oncology (SEOR) and the Spanish Society of Medical Physics (SEFM) for interstitial high-dose-rate (HDR) brachytherapy (BT) for gynaecologic malignancies. A nine-item survey—which included questions on experience with interstitial BT; indications and technique; applicator type; magnetic resonance imaging (MRI)-based planning; dose; fractionation schedule; and treatment planning—was sent to all radiation oncology departments (*n* = 174) in Spain in 2021. Responses were received from 36 centres (50% of all centres [*n* = 72] with a BT unit). The consensus-based recommendations presented here are based on a review of the available literature, professional experience among the group of experts, and in-person discussions held during the annual meeting of these two societies. We describe the results of the survey and the following: indications; contraindications; patient selection; description of applicators; role of imaging in planning; contouring; dose prescription; dosimetric reconstruction; optimisation; and dose indications for cancers of the cervix, vagina, and vulva. The various clinical scenarios in which interstitial BT is used in the treatment of gynaecological tumours are described in detail, including cervix intracavitary/interstitial hybrid HDR-BT; cervix perineal templates/freehand implants; primary vaginal malignancies/vaginal recurrences; and vulvar interstitial implants.

## Introduction

Brachytherapy (BT) plays a key role in the treatment of gynaecological malignancies, especially for cervical and endometrial cancer. Various BT techniques are available. However, interstitial brachytherapy (ISBT) is considered essential in the treatment of cancers of the cervix (early stage or locally-advanced), vagina, and vulva [1–3], and for the treatment of recurrent disease.

In the present document, we describe the clinical indications, applicators, and physics-related aspects (dosimetry, reconstruction, and prescription) defined by an expert group of radiation oncologists and medical physicists from the Spanish Brachytherapy Group (GEB; *Grupo Español de Braquiterapia*) and the Spanish Society of Medical Physics (SEFM). These recommendations are based a literature review, professional experience, and in-depth discussions held at the annual consensus meeting of the two societies, which took place on October, 22 2021 at the Catalan Institute of Oncology in Barcelona, Spain. The topic of this meeting was “Interstitial Gynaecological Brachytherapy”. Here we present our consensus-based recommendations.

Prior to the meeting, a brief, nine-item electronic survey was distributed to all radiation oncology departments in Spain and Portugal (*n* = 174) by the Spanish Society of Radiation Oncology (SEOR). In Spain, there are 124 radiation oncology departments; of these, 72 offer brachytherapy. Of these 72 centres, 43 are public, 17 private, eight public–private, and four are foundations [4]. A total of 36 complete surveys were returned, a response rate of 50% (half of the centres offering brachytherapy). The survey questions and responses are shown in Table 1.

**Table 1:** Survey questions and responses

Question	QUESTION	RESPONSE^1^
1	Do you usually employ interstitial brachytherapy?	Yes: 24 No: 12
2	In which tumours do you use an interstitial component?	Cervix: 23 (96%) Vagina: 21 (87.5%) Vulva: 18 (75%) Relapses: 21 (81%) *Response rates based on the 24 centres that perform interstitial BT
3	Do you use MRI for treatment planning?	Yes: 22 No: 5
4	Do you perform an image control before subsequent fractions for the same implant?	Yes: 16 No: 8 Only one fraction per each implant: 3
5	What kind of applicators can you use?	Fletcher: 1 Utrecht applicator: *18 Ring 8 Free hand needles 12 Plastic tubes 13 MUPIT * 9 Monoinstitutional perineal template: 5 Venezia applicator: * 6 3D individualised templates: 3 Fletcher + Tulip ** 2 Syed Neblet (Alfa Omega services, Bellflower CA) 1 MAC **1 Template Kelowna *** 1
6	What dose rate do you use?	HDR: 27 PDR: 2
7	What RT-BT scheme do you use?	Combination of RT and BT as a boost 26 Exclusive BT as reirradiation method 16 Exclusive BT depending on age and other factors 1 Exclusively BT in very early tumours 1
8	What RT-BT scheme do you use? Total dose and fractionation (specify if this is used as a boost or exclusive BT)	Boost: 14 centres 4 fr of 7 Gy in 2 implants 3 centres 4 fractions of 4-6 Gy, 6 or 6.5 Gy, 1 centre 6 fractions of 4.25 Gy, 1 centre 5–6 fractions of 3.5 Gy Relapses and exclusive BT: 1 centre 9 fractions of 4.5 Gy 1 centre 9 fractions of 3–3.5 Gy) 1 centre 8 fr 4 Gy 1 centre 8 fr 5 Gy 1 centre 6 fr of 6 Gy Centre applied 3 fractions of 7 Gy Vaginal primary cancer 1 centre 5–6 fractions of 4–5 Gy Vulva relapses RTE + 3 fractions of 7 Gy; Vaginal relapses RTE + 4 fractions of 3.5 Gy 1 centre 5 fractions of 5 Gy
9	Planning system used	Modified Manchester System (pear): 10 centres Inverse planning: 13 Paris System: 9 D90 HRCTV: 1 SEFM recommendations: 1 “conformal optimisation”: 1

*BT* brachytherapy, *HDR* high-dose rate, *RT* radiotherapy

^*^Elekta. Stockholm, Sweden

^**^Eckert & Ziegler Bebig, Germany

^***^Varian Medical Systems, (Palo Alto, USA)

^1^More than one response was possible for some items

## Indications/patient selection/contraindications

### Cervix intracavitary/interstitial hybrid HDR- BT

Image-guided brachytherapy (IGBT), in conjunction with external beam radiotherapy (EBRT) and concurrent chemotherapy, is the current standard treatment for cervical cancer.

The EMBRACE I study was performed to evaluate the role of IGBT in locally-advanced cervical cancer. This large, prospective study (*n* = 1341 patients) offers the highest level of evidence available at present [1]. Importantly, the results of the study validated the GEC-ESTRO and ICRU (International Commission on Radiation Units and Measurements) recommendations [5]. At a median follow-up of 51 months (interquartile range, 20–64), the actuarial 5-year local control rate was 92 (95% confidence interval: 90–93). EMBRACE I showed an absolute improvement of 14–17% in both local and pelvic control in patients with stage IIIB disease (FIGO) compared to previously reports [1]. This result is similar to that achieved with ISBT. Moreover, compared to previous studies with a similar stage distribution, the 5-year overall survival (OS) rate in EMBRACE I was superior (74 vs 67%) [1].

In cervical cancer, ISBT is indicated for stages IIB-III and IVA. An analysis of the retroEMBRACE study (a retrospective study of patients treated with IGBT based on computed tomography [CT] or magnetic resonance imaging [MRI] before initiation of the EMBRACE study) [6] showed that the patients that benefitted the most were those with large volume, high-risk clinical target volume (HR-CTV) at the time of BT. Local control in patients with HR-CTV > 30 cc was 10% higher for ISBT than for intracavitary BT alone [7], without any increase in late urinary or gastrointestinal toxicity.

The use of ISBT has grown substantially in recent years. For example, in the retroEMBRACE study, 23% of patients were treated with intracavitary or interstitial BT, while up to 43% of patients in EMBRACE 1 received ISBT.

### Cervix perineal templates/freehand implants

The potential coverage allowed by intracavitary/interstitial hybrid applicators is scant if any of the following are present:

- Medial or distal parametrial extension (up to pelvic wall)
- Unresponsive bulky disease
- Cervical tumours with vaginal extension to the middle or lower third
- Bladder or rectal involvement (stage IV)

Or in certain clinical situations, as follows:

- Cervical cancer in patients who are not suitable for an intrauterine component due to unfavourable topography
- Presence of poor geometric conditions: very narrow vaginas
- Previous history of total/subtotal hysterectomy where the gynaecological tandem cannot be used

In these cases, it is recommended to add a larger interstitial component [8]. The implant can be performed using transperineal templates or the “freehand” technique, with guided placement and planning by transrectal ultrasound (US), CT, or MRI [8–14]. Only a limited number of institutions perform interstitial perineal implants, possibly due to the invasive nature of the implants, insufficient experience with the technique, and/or due to the scant literature [15, 16]. Nonetheless, now that MRI is available for BT planning, and considering the need to provide better coverage of locally-advanced tumours in which conventional applicators (including hybrids with an interstitial component) do not allow offer good coverage, it, it is essential that patients have access to this technique when indicated.

### Primary vaginal malignancies/ vaginal recurrences

Primary vaginal cancer is a rare cancer, accounting for only 3% of all gynaecological malignancies. Due to its rarity, there is a notable lack of data on the optimal therapeutic management of this cancer, which represents a major challenge to improving treatment.

Historically, surgery was the treatment of choice for primary vaginal cancer. However, due to the need for an extensive resection, organ sparing was not possible, leading to severe morbidity with a negative impact on quality of life [17, 18]. Data from the historical series at centres where surgery was the standard treatment show 5-year OS rates ranging from 47 to 74%, with a median OS for stage I and stage II disease of 82 and 53%, respectively [19–21]*.* Given these data, there is wide agreement that surgery is the technique of choice in small tumours (< 2 cm) that are limited to the upper third of the vagina (17). Importantly, treatment with definitive EBRT or even BT alone has shown very good results in terms of local control and disease-specific survival, ranging from 83 to 100% [22, 23].

Since primary vaginal cancer is etiologically similar to cervical cancer, the same treatment strategies were implemented, which is why an organ-sparing approach consisting of radiochemotherapy followed by BT became the standard treatment in primary vaginal cancer. A large study based on the SEER (Surveillance, Epidemiology, and End Results) database found that the median OS in women who received BT was almost twice as long as those who received EBRT alone (6.1 vs. 3.6 years). Moreover, the addition of BT reduced the risk of death by 13%. On the multivariate analysis, BT was an independent predictor of survival [24].

The introduction of MRI-based IGBT in the treatment of cervical cancer allowed for dose escalation, which resulted in better local control rates and reduced morbidity [6, 25, 26]. In vaginal cancer, a few studies have reported 2D-radiograph-based BT outcomes, with good local control, especially in stage T1 disease [22–24]. Several small, single institutional series have introduced the terms of IGBT in vaginal cancer, showing encouraging results, with 2-year local control rates ranging from 82 to 93% with less morbidity than previous studies [27, 28].

In recent years, the Gynaecological Working Group of the Groupe Européen de Curiethérapie and the European Society for Radiotherapy and Oncology (GYN GEC–ESTRO) introduced the terms and target concept for IGBT in vaginal cancer [2]. Recently, that task group conducted a retrospective, multicentre study involving 148 patients with primary vaginal cancer treated with IGBT, showing a good local control rate (83%), with especially strong results in large advanced stage tumours (T3 and T4a) compared to previous reports [32, 33]. These results, although preliminary, support the role of IGBT in primary vaginal cancer based on the good local control rates in large tumours with less morbidity due to better sparing of organs at risk (OAR).

### Vulvar cancer

Vulvar cancer is rare, accounting for approximately 4% of all gynaecologic malignancies worldwide [34]. Although primary surgery (radical excision/vulvectomy with selective sentinel node biopsy and/or bilateral inguinofemoral lymphadenectomy) is the cornerstone of treatment for this cancer especially for early-stage disease [34–36], recurrence rates are high. The main factors influencing local recurrence are nodal involvement and insufficient surgical margins [37–39]; however, other factors may also contribute to increased risk of recurrence, including stromal invasion [39], lymphovascular [40] and/or perineural invasion [41], tumour size, and the presence of associated preneoplastic lesions and human papillomavirus [42, 43].

In locally-advanced vulvar cancer, the standard treatment—radical surgery—is not always feasible due to the difficulty of achieving clear surgical margins without performing mutilating surgery and/or because the lymph nodes are fixed to the fascia, muscle, or vascular structures. In these cases, the treatment of choice is definitive radiotherapy with or without neoadjuvant chemotherapy [44–50].

BT is indicated for the treatment of vulvar cancer in three clinical scenarios [3, 51–53], as follows: (1) postoperative adjuvant BT for patients with early-stage disease who have unfavourable histological prognostic factors. In this case, treatment options are definitive BT or BT combined with EBRT; (2) boost BT to the primary tumour after EBRT in locally-advanced vulvar tumours not suitable for upfront surgery; (3) local recurrence after primary surgery or previous irradiation.

## Applicators

### Intracavitary/interstitial hybrid HDR-BT

Intracavitary applicators consist of an intrauterine tube (IUT) and the vaginal component (ovoid, ring, or cylinder) to which the interstitial part can be attached in different ways (see below). The various commercially-available applicators are described below.

### Ovoids, intrauterine tube and interstitial needles

#### Elekta (Stockholm, Sweden)

- Utrecht applicator

The original Utrecht applicator is composed of two ovoids and an IUT (4 or 6 mm in diameter). In addition, up to five flexible plastic catheters measuring 294 mm in length with blunt/round tips or sharp tips can be placed in each ovoid, two in the central part to complete the effect of the IUT and three laterally to extend to the parametrial level with an output angulation of 15º and 25º. The IUT has a unique length with a cervical stop where the ovoids hook, thus allowing the applicator to be adapted to the hysterometry of each patient.

- Geneva applicator

This applicator is superior to the older Utrecht-type applicators, especially for patients with smaller anatomies. This modular applicator has a greater size range of ovoid tubes, with a fixed diameter IUT in six different lengths and three angulations. It allows for the placement of at least five needles per ovoid, and more needles can be inserted in larger diameter ovoids. In addition, a central interstitial needle can be added to expand treatment options after hysterectomy. The different components are easily assembled due to the new clip-on system.

## Varian medical systems (Palo Alto, USA)

The interstitial component is coupled to a Fletcher-type titanium applicator with a 3 mm IUT with different lengths and a 30º angulation. The pairs of ovoids have holes that act as a guide or template for the interstitial component, allowing for insertion of sharp or blunt tip needles. The needles are 2 mm in diameter and 320 mm in length, parallel to the IUT. The ovoids allow for the placement of 4, 6 or 8 needles depending on the size of the ovoid.

### Ring, intrauterine tube, and interstitial needles

#### Elekta (Stockholm, Sweden)

- Vienna applicator

This applicator has capacity for up to seven titanium needles (26 mm ring), and up to nine needles in the larger diameter (30 mm and 34 mm) rings. Needles can be plastic or rigid metallic with an angulation of 60º to the vaginal axis. The rigid needles (1.9 mm in diameter and 240 mm in length) are pre-curved with penetration distances of 30 mm, 40 mm and 50 mm, and are also located parallel to the IUT of fixed lengths.

- Vienna II applicator

The Vienna II ring applicator was developed to treat patients with residual distal parametrial disease that cannot be adequately covered by hybrid ovoid or ring applicators [54]. The Vienna II is the same ring applicator as Vienna, but with an additional piece (a hood that is covered to the vaginal ring). This extra piece serves to help guide interstitial needles with an oblique direction that is 20º relative to the IUT.

- Venezia applicator

This applicator consists of an IUT, two interstitial semicircular tubes forming an easy-to-assemble ring, two vaginal capsules that adhere to the corresponding semicircular tube, and a perineal template through which the needles are inserted in parallel from the perineum (freehand) to the parametrium. The perineal bar is a tool used to fix the applicator to the patient. This hybrid applicator allows users to insert interstitial needles in parallel or obliquely (12º) to the intrauterine tube depending on the patient’s anatomy. This applicator has the capacity to insert up to 134 6F (2 mm) plastic needles (length: 294 mm), both in parallel and divergently angled to the IUT. Each crescent can accommodate up to six needles, and 122 in the perineal insole. This design offers an important advantage for dose distribution in BT in complex volumes due to the large number of channels, their separation and their orientation, allowing for the delivery of an optimised dosimetric coverage in terms of compliance with the HR-CTV, which can reduce doses to the OARs.

## Varian Medical Systems (Palo Alto, USA)

This applicator consists of intrauterine tubes of different lengths with a ring angulation of 60º and 90º to the vaginal axis. The applicator incorporates a sheath that increases the mucosal source distance while also allowing for the insertion of interstitial vectors. This applicator has the capacity for 16 plastic needles, 2 mm in diameter and 320 mm in length, parallel to the IUT.

## Eckert and Ziegler Bebig (Germany)

This applicator consists of three intrauterine tubes with different angulations and three different lengths. It has a thinner IUT (3.5 cm in diameter) with a length of 60 mm. The applicator can attach a 30 mm ring with a double channel, allowing for the incorporation of a rectal retractor. This applicator has the capacity for up to eight plastic needles (diameter: 1.7 mm, length: 300 mm) parallel to the IUT or with an angle of 5º.

The manufacturer (Eckert & Ziegler Bebig) markets the “Tulip Applicator Family”, which allows clinicians to adapt the endocavitary applicators to a guide mold, which allows for the guided placement of plastic needles in different central and lateral positions at various different angles. This system can be used with HDR afterloaders from other companies. The capsules are individual consumables and cannot be used for more than 24 h.

### Perineal templates

The classic manufactured applicators, which were designed for CT scans, are being modified for use with MRI. There are perineal templates for use with rigid needles (aluminium or titanium) that minimise implant deviation, which is important considering the long path that the needles must travel from the guide in the perineum (minimum: 10 cm). This long distance can be an inconvenience with plastic catheters, especially if the objective is to add the necessary obliquity to ensure coverage of distal parametrial disease.

#### CT compatible applicators

- Martinez Universal Perineal Interstitial Template (MUPIT)

This template is designed for gynaecological, prostate, anorectal, and perineal implants. The MUPIT consists of a double perineal template, a vaginal and rectal cylinder, and hollow 17-gauge guides separated by 6–10 mm. It has a total of 111 holes, which permit straight or angled vectors in horizontal planes, perpendicular to the plane of the perineal template. These are angled distally 14º, achieving adequate implantation of the parametria and greater lateral coverage of the CTV. The metal needles vary in length (range, 14–20 cm) and are held with stoppers to prevent cranio-caudal displacement during the procedure. A second template is used to strengthen vector fixation. The disadvantages include the lack of an intrauterine tube and the need for CT-based planning.

- Syed-Neblett applicator

The modified Syed-Neblett device (Alfa Omega services, Bellflower CA) is based on the same principle as the original applicator and is only compatible with CT. It consists of a perineal template and a vaginal obturator with hollow 17-gauge guides of various lengths that allow users to combine interstitial sources and a vaginal cylinder with or without an IUT.

#### MRI-compatible applicators

- Venezia Applicator

See description above.

- MAC applicator

The MAC (acronym for the “Mick-Alektiar-Cohen” collaboration) applicator from Eckert & Ziegler Bebig (Germany), consists of a cylinder + IUT, is compatible with MRI and can be used with plastic needles of various diameters up to 2 mm. It has 36 concentric channels for the placement of unilateral or bilateral needles in parametria and can be used with a 0º or 30º IUT.

- Kelowna template

Varian Medical Systems has developed the Kelowna gynaecological templates for interstitial implants, with a universal cylinder as a vaginal obturator. A 25 mm central hole in the template allows for the insertion of an ultrasound probe to guide the placement of needles of various sizes and lengths (113, 200, or 320 mm), whose tips can be sharp or blunt. The needles can be made of polyetheretherketone (PEEK), steel, or titanium.

**Fig. 1:**
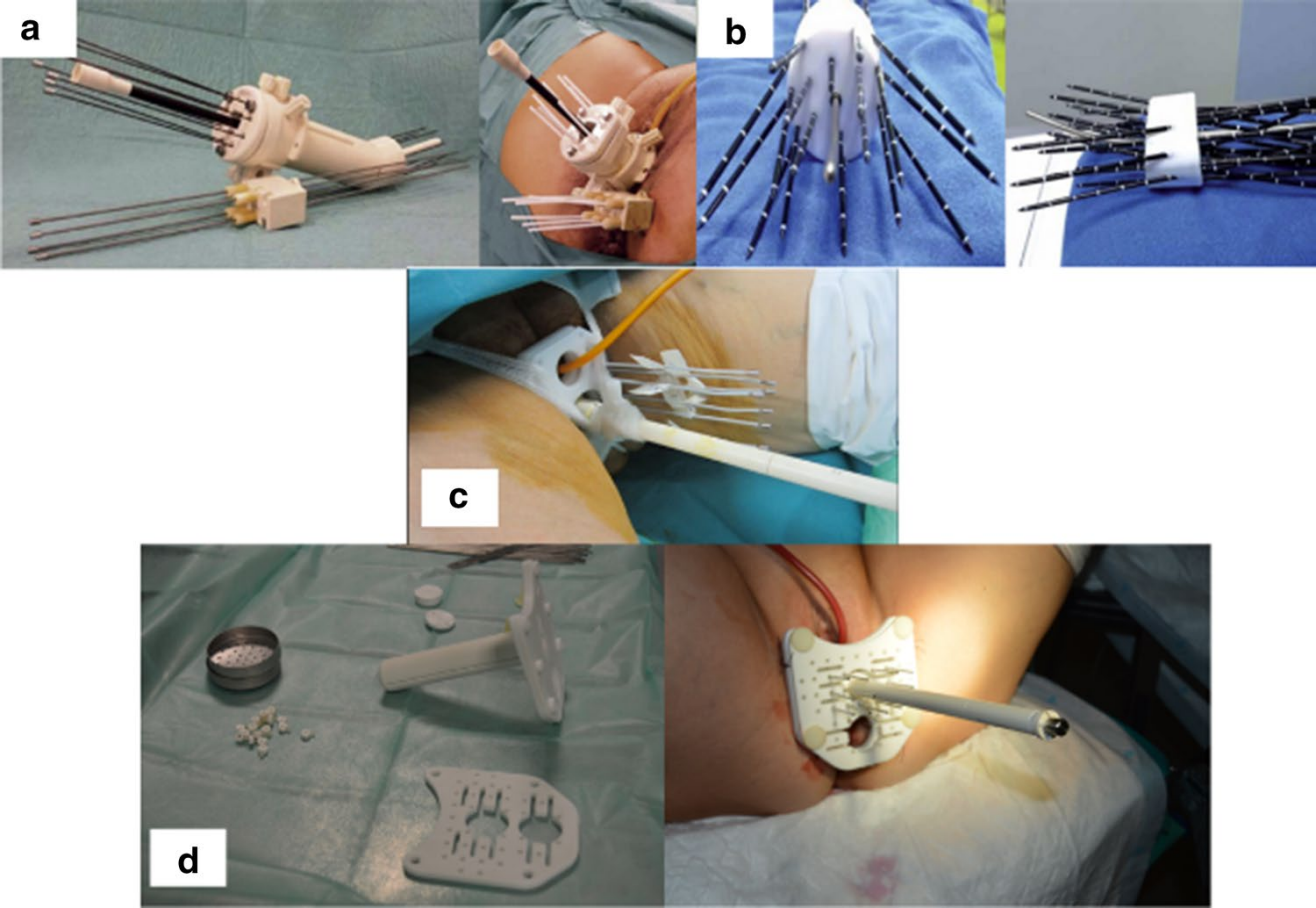
**a** Antoine Lacassagne Cancer Centre. Niza. **b** Medanta applicator AEOLO. **c** Pamplona applicator **d** Benidorm template

Several teams with extensive experience in perineal ISBT have developed their own (i.e., non-commercial) MRI-compatible applicators that include an IUT. These applicators use rigid titanium or plastic, straight or angled needles, and can provide distal parametrial coverage through the adaptation of commercially-available IUTs. In Spain, two well-known prototypes are the Benidorm and Pamplona templates (Fig. 1).

#### 3D printing for the individualised treatment of cervical carcinoma

In recent years, notable advances have taken place in terms of the development of three-dimensional (3D) printers for medical supplies. These printers allow clinicians to design patient-specific applicators adapted to the patient's individual anatomy and tumour response. This allows for individualised solutions not available with standard commercial applicators. It important to note that these applicators must be produced in a healthcare environment to ensure their suitability for clinical use in patients.

**Fig. 2:**
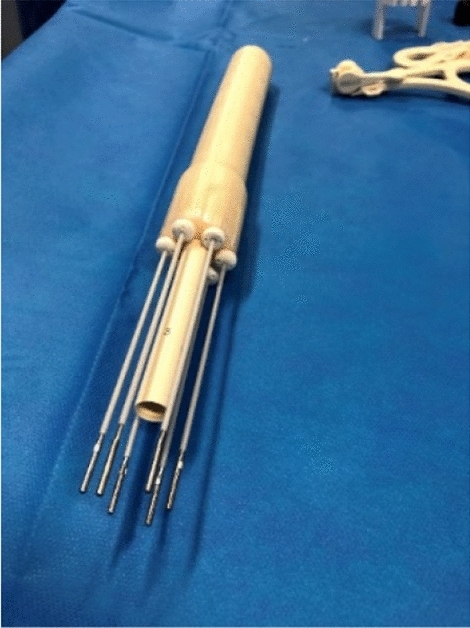
3D printed applicator. Courtesy of Dr. Elena Villafranca

The Navarra Hospital Complex developed a 3D printed endocavitary-interstitial applicator to treat the entire length of the vagina irregularly and to insert parametrial needles. This applicator is shown below in Fig. 2.

#### Interstitial BT without perineal template (“freehand”)

Interstitial implants can also be performed freehand (i.e., without the use of perineal templates) guided by vaginal and rectal touch, ultrasound or laparoscopy.

The disadvantage of freehand needle insertion is the complexity of the technique. In addition, msince the geometry is not predefined, it is very user-dependent, which could lead to imprecise and unstable positioning, poor reproducibility, and potential loss of parallelism between the needles.

## Technique

### Perineal templates

Perineal implants with templates present some differences compared to intracavitary/hybrid HDR-BT applicators. Before performing this technique, the first step is to determine exactly how many needles are needed, and their position, obliquity, and depth. Implant displacement must be taken into account when switching the patient from the gynaecological to the supine position, and when repositioning the needles to the cranio-caudal direction, as this can lead to insufficient coverage of the CTV. During repositioning, it is important to consider this movement when inserting the needles and also the needle offset, which will indicate the first position of the source and the start of irradiation.

Ideally, needle insertion should be guided by ultrasound, marking the gross tumour volume (GTV) with clips or fiducial markers. The bladder should be filled with radiopaque contrast to allow for bladder visualisation to reduce the risk of perforation.

The templates have a central vaginal cylinder, which maintains the geometric rigidity of the needles. However, one of the drawbacks of the commercial templates is that only one cylinder size is available (length: 13 cm; diameter: 3 cm), which is problematic in patients with a narrow, short, or poorly distensible vagina. If the vaginal cylinder cannot be placed, it can serve as a guide to place the vectors, and be removed later.

The template is sutured to the perineum. The needles have a stop to prevent displacement. Perineal implants are usually performed only once and then left in place for several days to administer several fractions (delivered twice daily). For this reason, preventive measures are necessary (i.e., anti-bedsore techniques, analgesia, antithrombotics, and antibiotics). The patient cannot move nor raise the headrest above 30º. It is essential that the staff receive specialised training to avoid complications while the implant remains in place.

### Interstitial vulvar implants

This inpatient procedure requires hospital admission. Bowel preparation prior to the implant is recommended. The procedure is performed in the operating room with the patient in the lithotomy position under general or spinal anaesthesia. Bladder catheterisation is recommended. A thorough clinical examination under anaesthesia with disease mapping is recommended prior to implantation. Before planning, it is essential to consider the implant design; if possible, the tubes should be inserted anterior-posteriorly, rather than transversally, to prevent the tube from bending when the legs are lowered from the lithotomy position.

Interstitial implants can be performed with rigid needles or plastic tubes, with the latter being more comfortable for the patient. The procedure is usually performed freehand. The target volume encompasses the GTV—or the tumour bed in case of postoperative BT—plus a safety margin of 5–15 mm. Vector needles are inserted parallel to the labia or the vulvectomy scar in one or two planes to cover the residual tumour size in all three dimensions. Spacing should be guided by the Paris system rules. The distance between needles ranges from 10 to 15 mm. Special care is needed to prevent hot spots (200%). If necessary, extra catheters can be added to the surface of the tumour to ensure adequate coverage. The needles can be replaced by plastic catheters, fixed with buttons to the skin at the entry and exit points to ensure their stability, and can be combined with a vaginal cylinder or vaginal packing as a stay.

In deep tumours involving the vagina, urethra, paravaginal or paraurethral space, a combined technique consisting of a vaginal cylinder and rigid needles with or without a perineal template should be used. In these cases, vaginal and endorectal ultrasound may be useful to guide the placement of rigid needles.

Analgesics, prophylactic antibiotics, and anti-oedema measures are recommended throughout the hospital stay until treatment finalisation. Implant removal should be performed carefully after treatment completion, using all necessary aseptic precautions. After implant remove, the patient can be discharged.

## Treatment planning

### Planning images

The type of applicator is usually defined at diagnosis, based on clinical examination and previous MRI. Locally-advanced tumours may have different growth patterns: expansive or infiltrative and responsive, and may produce anatomical changes and changes in the adjacent topography. Whenever possible, MRI should be performed prior to BT to allow for selection of the applicator that offers the most complete coverage. The routine use of preplanning will facilitate the BT procedure in locally-advanced cervical tumours by offering the potential to obtain a geometrically optimal implant, although these are not currently available. The main drawbacks to this approach are the anatomical changes that occur when placing the IUT in the implant and straightening the uterus, and the scant studies on this procedure [55, 56].

The gold standard imaging technique for cervical carcinoma is MRI, which is used for diagnosis, to assess treatment response, and for BT treatment planning. In 2012, the GEC-ESTRO published recommendations (4th edition) on the use of MRI, which included recommendation on how to prepare patients for the scan, as well as technical aspects and image acquisition protocols [57]. Although these guidelines can serve as a baseline, each department must develop, in collaboration with the radiology department, its own criteria for MRI [58]. In most centres, the MRI units are either 1.5 or 3 T, with 1.5 T MRI being the most common. Ideally, the duration of the imaging study should as short as possible to minimize movement. Intravenous contrast or intracavitary coils are not necessary. Although MRI is superior to CT in cervical carcinoma, not all radiotherapy departments have access to this imaging modality nor the capacity to perform MRI-based dosimetry. Moreover, MRI is more expensive than CT and involves a major learning curve, which explains why CT continues to be used for BT planning in many departments around the world.

MRI is also recommended for target volume delineation when using ISBT to treat vulvar tumours. However, due to the complexity of reconstruction of the plastic needles in MRI images, fusion with CT is often required. Image fusion must be highly precise, and CT scans should be acquired with the patient in the supine position (slice thickness: 1–3 mm). To help in the contouring process, a thin wire may be placed on the tumour edge prior to acquisition (this wire may be used for target contouring in each slice).

### Contouring/prescription

#### Intracavitary/interstitial hybrid HDR-BT

The contouring recommendations proposed by the GEC-ESTRO have been accepted by the main brachytherapy societies, including the American Brachytherapy Society (ABS) and the GEB. Contouring of the following structures is recommended: (1) GTV: visible macroscopic tumour at the time of BT, detected by physical examination or as a bright image on the T2 MRI sequence; (2) HR-CTV: the high-risk tumour volume includes the GTV, the entire cervix, and any microscopic residual disease in the initial tumour location (parametrium, vagina), which are visible on the T2 MRI sequence as residual gray areas; (3) IR-CT: the intermediate-risk tumour volume, defined as the initial tumour location. This volume is calculated by expanding the HR-CTV with a 5–10 mm margin in all directions, depending on tumour response and limited by the risk organs; 4) OARs: rectum, sigmoid, bladder and intestinal loops should be contoured.

- Rectum: This should be contoured as a complete organ from the anal margin to the peritoneal reflection.
- Sigma: This should be contoured as a complete organ from the peritoneal reflection to two cm above the fundus.
- Bladder: This is contoured as a complete organ, marked by the end of the bladder neck after the balloon is no longer visible from the bladder catheter.
- Intestinal loops: These loops should be contoured at least in the 2-cm area surrounding the HR-CTV.

#### Perineal templates

Perineal implants have special characteristics, so we cannot always apply the published recommendations for intracavitary/hybrid implants. The process involves a single implant that covers both the disease at diagnosis (IR-CTV) and the disease at the time of BT (GTV and HR-CTV), in which treatment volumes are often much greater than usual, especially in cases with involvement of the distal parametrium or the entire vagina. Planning is usually CT-based, although this makes it more difficult to define the tumour, cervix, GTV, and OARs. In implants that use MRI-compatible applicators, the CTVs are smaller, thus higher doses can be administered with less toxicity. Although perforation of healthy organs is not uncommon, needle removal is not indicated since there is no increase in toxicity if appropriate measures are applied (e.g., antibiotics) and these positions are left unactivated at the time of planning [13, 16].

#### Primary vaginal malignancies/vaginal recurrences

In the last decade, several different multi-institutional consensus statements and guidelines have been developed to define BT volumes in vaginal cancer [29, 30, 32, 33, 59]. Volume definition in BT has evolved from the use of orthogonal plaques for the treatment plan to MRI-guided imaging. As a result, the various guidelines and consensus statements differ in terms of imaging and CTV definition [59].

In 2012, the ABS published guidelines and recommendations for ISBT for vaginal cancer (primary tumours and recurrent disease) [59]. In those guidelines, the CTV was defined as the residual tumour on MRI at the time of BT plus the cervix and the whole vagina. As those guidelines underscore, either intracavitary or interstitial BT may be used, depending on the tumour extension, thickness (< or > 5 mm), location, and morphology [59].

For primary vaginal cancer, the GYN GEC-ESTRO group has adopted the validated target concepts used for cervical cancer. In 2019, the task group published the first recommendations and target concepts for IGBT in primary vaginal cancer, pending evaluation in a prospective, multicentre study. MRI and gynaecological examination at the time of BT play an important role in defining target volumes for IGBT.

Based on concepts learned in the treatment of cervical cancer, the following volumes are defined:

- GTV-T_res_: gross residual tumour at time of BT on clinical examination and/or imaging
- CTV-T_HR_: high-risk clinical target volume, including the residual GTV-T and areas with the presence of pathological tissues.
- CTV-T_IR_: intermediate-risk clinical target volume, including all significant microscopic disease adjacent to the HRCTV-T.

For the treatment of vaginal recurrences, several multi-institutional consensus statements have been published based on the target concepts previously defined by the GEC-ESTRO [72, 73]. In bulky vaginal tumours (target volume or OARs), the Canadian consensus recommends including the entire circumferential vagina in the CTV-T_HR_ at the GTV level. In addition, the CTV-T_IR_ should include the entire vaginal circumference at the level of the CTV-T_HR._ [60]. The Canadian consensus also adds a larger margin at the cranio-caudal level, following the recommendations of the GEC-ESTRO consensus. However, there is still some debate regarding tumours with adenocarcinoma histology, with many authors recommending inclusion of the entire vaginal length in the CTV-T_IR._

At present, work is ongoing to develop consensus terminology for dose prescription.

#### Interstitial vulvar BT

Preferably, the GTV should be delineated on MRI. In the adjuvant setting, if no macroscopic disease is present, then no GTV BT is delimited. GTV BT includes postoperative residual vulvar/perineal disease detected on imaging and/or physical examination. In locally-advanced vulvar cancer, the GTV BT includes vulvar/perineal disease detected on imaging/physical examination. In both residual vulvar/perineal disease and in locally-advanced vulvar cancer, the macroscopic volume should be contoured if BT is administered as a boost after EBRT; for primary BT, the tumour volume at diagnosis should be contoured.

The target volume encompasses the GTV (or the tumour bed in case of postoperative BT) as well changes detected on MRI if EBRT is administered prior to BT. The target volume also includes all suspicious findings on physical examination with a 10–15 mm margin (excluding certain structures [e.g., urethra, anus]). The larger margin can be applied in the same direction as the tubes.

According to the Paris system, dose prescription should be at the reference isodose (85% of the minimal dose rate between planes). Doses should be reported after conversion into radiobiologically-weighted dose equivalent of 2 Gy/fraction (α/β 10 Gy for the tumour, half-time of 1.5 h), EQD2 Gy [3, 51–53].

### Reconstruction

#### Intracavitary/interstitial hybrid HDR-BT

In recent years, BT techniques have improved substantially. Probably the most notable improvement is the potential to individualise treatment at any point in the process. A wide variety of applicators are available, thus covering the diversity of different tumour anatomies. In this regard, the recent addition of the interstitial component to standard applicators is an especially notable improvement. In addition, wider access to volumetric imaging techniques (e.g., CT, MRI, and US) has made it possible to adapt dosimetry to anatomical volumes, thus obviating the older points-based dosimetric techniques. These two changes have, in turn, altered the paradigm of applicator reconstruction and dosimetric optimisation. Below we summarize the recommendations of the GEB-SEFM group for both of these points.

A wide variety of applicator and imaging modality combinations are available (plastic or metal applicators, CT, MRI or US imaging). As a result, it is necessary to approach each option differently. One of the great challenges for medical physicists is reconstruction of the applicators, which requires proper commissioning and quality control of the applicators (including the different imaging modalities used and the corresponding dummies). These new forms of work introduce new uncertainties that must be considered. In this regard, we recommend that clinicians read the studies by Hellebust et al. and Tanderup et al. [61–63].

The accuracy of applicator reconstruction is conditioned by the image acquisition parameters, especially the slice thickness. Currently, the maximum recommended slice thickness is ≤ 5 mm (64). However, keeping in mind this limitation, the individual centre may choose to acquire images (both CT and MRI) with smaller thicknesses.

When using CT images, it is important to note the risk of large artifacts produced by applicator dummies and markers, which could hinder accurate contouring of the volumes of interest (VOI; target volumes and OARs). Usually, the applicator channel is visible on CT images, so we may consider omitting dummies, referring the dwell positions to the visible tip and walls of the applicator. It is also recommended to determine whether CT image reconstruction can be done through the application of iterative techniques and artifact reduction.

In some cases, when working with MRI, there is an added difficulty: the need for a commercially-available dummy. Due to lack of availability, most centres must use customised solutions designed in-house, such as tubes filled with fluids (e.g., saline, paraffin, etc.) that are compatible with and visible on MRI.

The diversity of MRI sequences must also be considered. The best MRI sequence to visualise tumour tissues is T2, but this is not the most suitable sequence for applicator reconstruction, for which T1 or CT are better. In this situation, registration of the different image sequences can be assessed. If this option is selected, it is essential to ensure that the registration is performed with a focus on the rigid part of the applicator instead of the VOIs.

Regardless of the chosen imaging modality, it is highly recommended to take advantage of the different views (axial, coronal, and sagittal). If different MRI sequences are used, it is important to verify that the patient has not moved between acquisitions; if so, this must be taken into account. The recommended view used to outline the VOIs should be the one preferred for applicator reconstruction, especially if there is any mismatch between the different views. For example, Tanderup et al. [64] recommended using a longitudinal plane to avoid the uncertainty caused by the slice thickness. In this image set, the distance from the centre of the axial slice to the channel centre can be measured.

In short, several different reconstruction options are available. These can be summarized as follows: (1) direct reconstruction on CT or T2 MRI slices, or (2) reconstruction on the support image (CT, T1 MRI, or other acquisition sequences) and registration between support and reference images. In recent years, some institutions have started using 3D-MRI acquisitions, which simplifies applicator reconstruction, even in T2-weighted series (by reducing slice thickness).

Finally, it is worth noting that several commercial vendors are developing automatic reconstruction methods, such as those based on MRI-tracking of a virtual source. When these tools become available, this will greatly facilitate catheter reconstruction, thus shortening the process. This is important given the most time-consuming task after applicator insertion is applicator reconstruction. Automating this process will likely reduce the risk of mistakes.

When all the catheters are visible on the image it is important to verify that the reconstructed channels are correctly assigned to the real channels of the applicator to avoid connection errors.

## Reconstruction tips

Due to the existence of various applicators and the possibilities offered by each planning system, the channel reconstruction procedure may vary. In general, it is recommended to use axes that can be modified by the user and whose orientation is parallel to the reconstruction channel.

Before starting channel reconstruction, it is advisable to perform a general review of the channel positions, verifying the distance between channels (for example, between ovoids) and/or expected angles between the IUT and the ring surface.

For rigid applicators, manual or applicator library reconstruction methods can be used. For curved and multi-channel applicators, it is highly recommended to use the libraries, since this reduces errors [61]. When using MRI, the reconstruction can be performed relative to the applicator surface if the source path has been adequately characterised in that way during commissioning.

Some applicators consist of several modules (e.g., two ovoids, or a ring and IUT); in these cases, the applicator library must allow each catheter to be manipulated separately if the user so desires. Catheter deformability is highly recommended.

If the applicator library is not available, an alternative is to create a plan library. In this case, the user must make a “perfect” reconstruction in the preferred image modality, where the source path is easily identifiable. Once the reconstruction has been made, some planning systems allow the reconstruction to be saved as a plan, which can then be imported for use with any patient. When importing a reconstruction, specific applicator points must be identified so that placement of the applicator in the images matches perfectly (as occurs in the applicator library).

By contrast, the interstitial needles must be directly reconstructed, since there are very few automatic reconstruction solutions in current planning systems. Most of these methods are based on the contrast differences that the channels produce with respect to the surrounding tissue, so they should work somewhat better with CT images, but not with MRI. When automatic methods are used, the reconstruction should be verified based on the information available from commissioning.

During direct reconstruction, the use of too many points can lead to an unrealistic “zig-zag” path, which is highly likely to produce undesirable deviations in dose distribution. When the canal has a rectilinear shape, which happens with standard applicator needles or metallic needles, it is better to utilize a reconstruction that has only two or three points.

The direction of treatment (i.e., whether the source stops are entering or exiting the applicator) must also be considered. The actual source position will not exactly match the positions indicated by the markers; the tension of the source cable will cause it to be attached to the external wall when the source enters the applicator during treatment and attached to the internal wall when it leaves.

Reconstruction of applicators that combine rigid and interstitial components will probably require a mix of approaches.

### Perineal templates

#### CT Based reconstruction

Traditionally, perineal templates for interstitial cervix BT were based on stainless-steel needles. In the mid-1980s, Martinez et al. developed the MUPIT applicator; however, that applicator was not MR-compatible due to the use of stainless-steel needles. This meant that reconstruction had to be performed on CT-based study sets, which unfortunately, results in poor soft tissue contrast. Due to this poor contrast, together with image artifacts caused by the metallic needles, target visualisation is poor. In turn, this impedes precise delineation of the PTV.

**Fig. 3:**
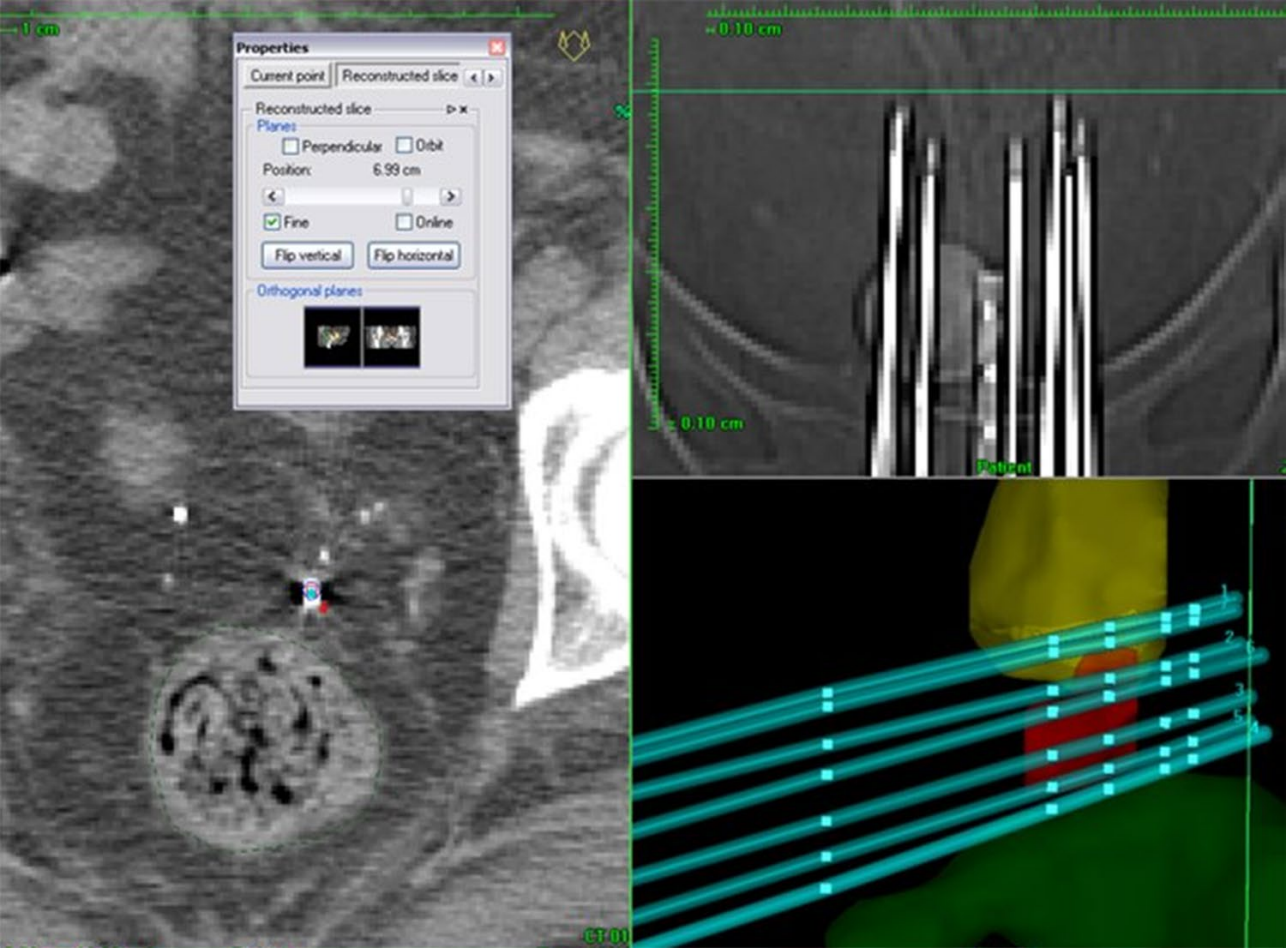
Use of scout view to determine the needle tip on CT images

In these cases, a preplanning CT study set is useful. First, a CT study set of the patient is acquired and the perineal template is superimposed to the axial CT images to select the most appropriate location of the needles to ensure optimal target coverage. The most difficult part of this reconstruction is to precisely determine the tip location, and thus the first source dwell position. The accuracy of this determination is directly related to the slice thickness in the study set. Some treatment planning systems (TPS) have tools to perform “fine” navigation between slices. The scout view may be useful to precisely determine the needle tip localisation. In the cranio-caudal direction, the scout view has no deformation, thus allowing the user to visualise the needle tip to correlate it with the corresponding axial image (Fig. 3).

#### MRI-based reconstruction

The use of MRI images for perineal ISBT procedures has several major advantages, including good soft tissue contrast and spatial resolution (3D sequences with isotropic voxel size ≈ 1 mm). Nevertheless, several issues must be considered when using these images [58]. First, the applicators must be MRI compatible. Generally, perineal BT procedures are performed with titanium needles, which can be problematic in MRI images due to geometric distortion and magnetic susceptibility artifacts of the titanium needles. Nevertheless, several authors have developed strategies to overcome these drawbacks [65].

In addition, geometric distortion must be assessed for the same type of sequences used clinically to obtain images suitable for planning. Commercial MRI distortion phantoms are available for these purposes.

**Fig. 4:**
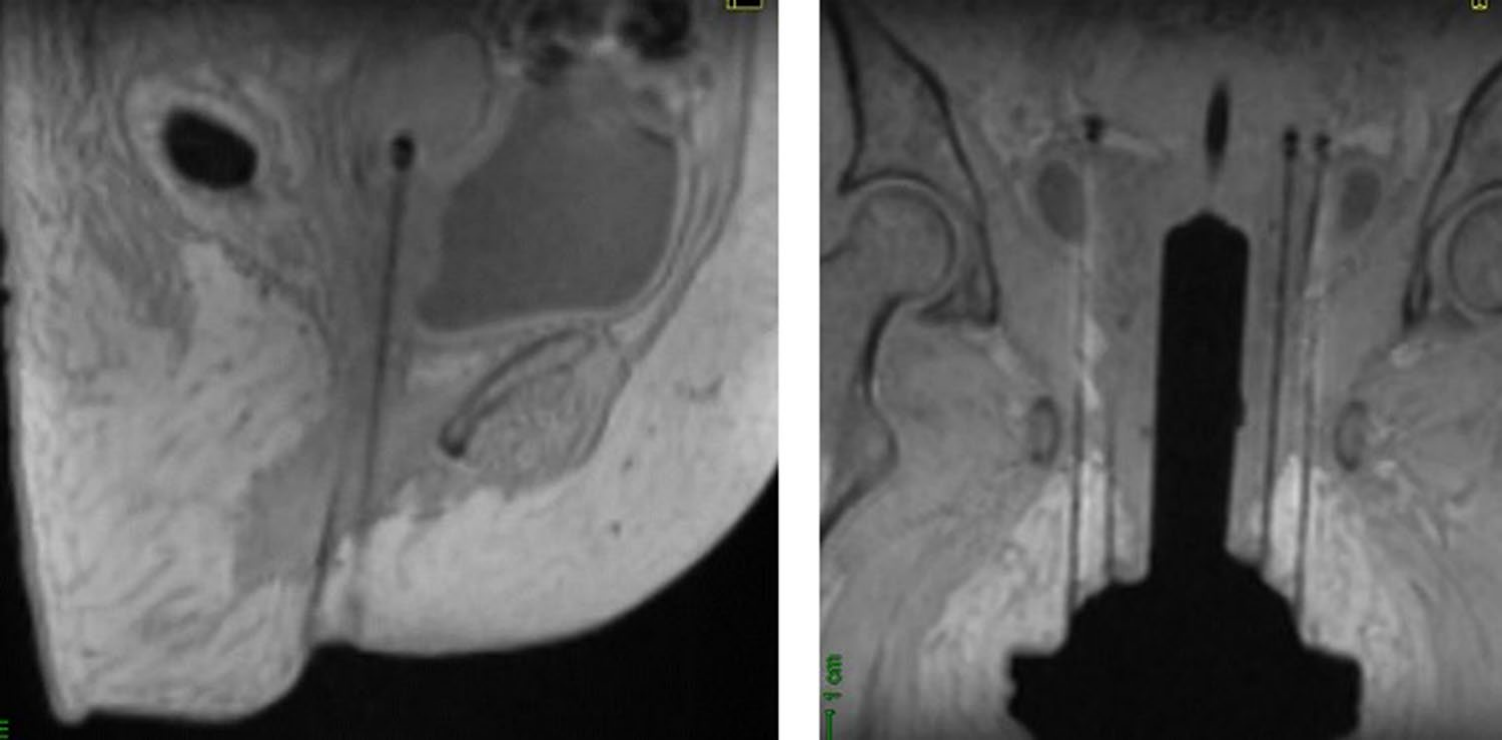
MRI images showing a “ballooning” artifact at the end of the titanium needle

**Fig. 5:**
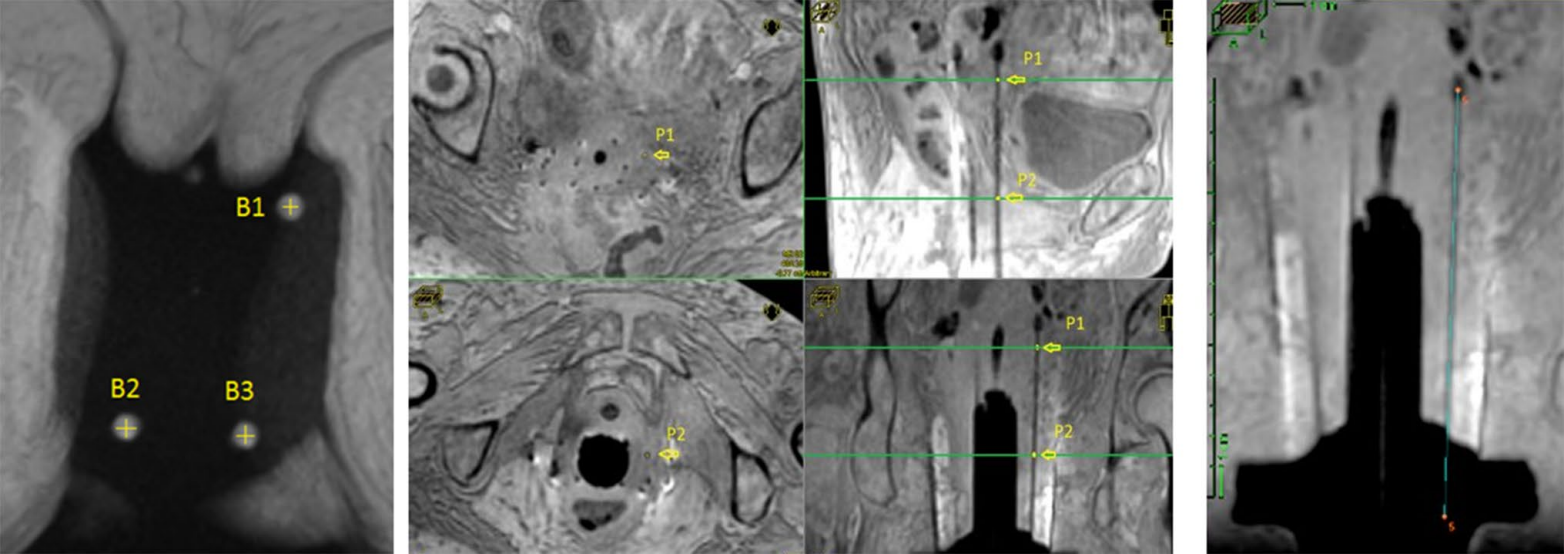
Reconstruction method for titanium needles based on MRI images with the aid of MRI markers (A-vitamin pellets)

The signal of titanium needles on MRI images shows a “ballooning” artifact at the tip of the needle, making it difficult to determine the exact distal dwell position (Fig. 4). However, there are many different solutions to solve this problem. For example, Haack et al. [66] identified the artifact on MR images and correlated it to the dwell position by performing a registration with a CT study set obtained during the commissioning process. Other authors [67] have suggested a method in which MRI markers (A-vitamin pellets)—which produce a high signal on MRI images—are embedded into the perineal template to identify a reference plane on these images. The direction and free length distance of the needles are used to obtain the needle tip coordinates (Fig. 5).

Rigid applicators commonly used in BT may be modelled into a TPS applicator library. Otal et al. [68] developed a method to incorporate interstitial components into the TPS gynaecologic rigid applicator library.

As mentioned above, a precise source path determination is required during the reconstruction process. In some situations, an additional series of images may be acquired—such as T1W, 3D-MRI or 3D Echo SPGR sequences—to improve applicator visibility. Registration accuracy between T2W and the other type of images must be assessed.

### Optimisation

#### Intracavitary/interstitial hybrid HDR-BT

Due to the rapid technological advances in the treatment of cervical cancer, currently there are no unified recommendations on how to optimise BT. The GEC-ESTRO gynaecology working group announced at the World Congress of Brachytherapy in 2021 that they intend to publish planning recommendations in 2022. However, pending publication of those guidelines, the criteria followed may vary from centre to centre, generally based on the centre’s experience together with published data from reference centres and other studies.

The protocols followed by Spanish radiation oncology centres are described below, as reflected in the paper published in 2018 by Perez-Calatayud et al. on behalf of SEFM [58].

Before discussing planning tips, it is important to mention that the participating centres in the study of SEFM agree that inverse optimisation should not routinely be used for this type of implant due to loss of control of the assigned times and because, in some situations, while the dosimetric parameters may be adequate, the implant may be excessively heterogeneous[58]. For these reasons, manual methods are usually recommended. The isodose lines in the three views of the implant should be reviewed.

- Activation of dwell positions

The step size should be based on the source size and activity. If the step size is too short, it could result in insufficient stopping times, which could increase uncertainty and ultimately cause deviations from the planned dosimetry.

The activation of positions should refer to the VOIs, encompassing the entire HR-CTV plus 5 mm. If any OARs are located near active positions, deactivation of those positions should be considered. Selection of the margins for activation and the final active positions must be confirmed by the radiation oncologist.

- Optimisation of dwell positions

This is the part of the planning process in which the greatest differences among centres can be observed. Two very different optimisation methods are described below for case in which a discrete number of needles are used. Later in this document, we describe other methods for use when the principal component of the implant is interstitial.

1. In this first method, points are generated on the surface of the HR-CTV and the plan is normalised to these points, initially without optimisation (i.e., all dwell times are equal). Next, the stopping times are modified through manual optimisation to meet the criteria established at the treatment centre (e.g., maximum volume allowed for 200% [V200%], V150%, rectal and bladder doses, and HR-CTV and CTV-IR coverage).

In the second method, planning is started with standard loading of the intracavitary component and normalisation to the A points (as established in the ABS 2012 recommendations) [59]. Stopping times are modified to meet the dosimetric criteria for OARs at the treating centre. Next, positions in the needles are activated inside the HR-CTV (taking into account potential OAR hotspots) and short times are assigned (1–4 s) in an effort to maintain the same time at all positions. According to the initial published recommendations, the total time assigned to the needles should be < 20% of the total treatment time; however, this restriction has lost importance over time, as the benefits of increasing the IS/IC time ratio has become increasingly clear [57].

Another point to keep in mind is that recommendations for the current dosimetric parameters have mostly been described for their evaluation in EQD2 (equivalent dose in 2 Gy fractions), by adding EBRT and BT dose. To our knowledge, at present (October 2022), none of the currently available TPS allow for dose evaluation in terms of EQD2 (nor for adding doses with EBRT). Consequently, medical physicists must use spreadsheets to carry out this dose assessment requirement.

Finally, it is important to emphasise that optimisation must be carried out in close collaboration with the radiation oncologist, especially evaluation of the EQD2 and dose distribution.

#### Perineal templates

- Activation of dwell positions

Activated dwell positions must assure adequate CTV coverage, which is why dwell positions 3–5 mm beyond the CTV are often activated. In this regard, it is important to keep in mind that the metallic needles (titanium or stainless steel) have a blind end about 1 cm in lenght, which should be considered when inserting the needles. Many planning systems have tools to automatically activate dwell positions to cover the CTV with an adequate margin. The source step is 2–3 mm.

Three primary strategies are available for optimisation: (1) inverse planning, (2) point-based optimisation, and (3) geometric optimisation.

The experience with the inverse planning modules included in the TPS in these types of implants, dose distributions obtained via inverse planning are similar to those obtained with other methods in terms of target coverage, but more heterogeneous.

**Fig. 6:**
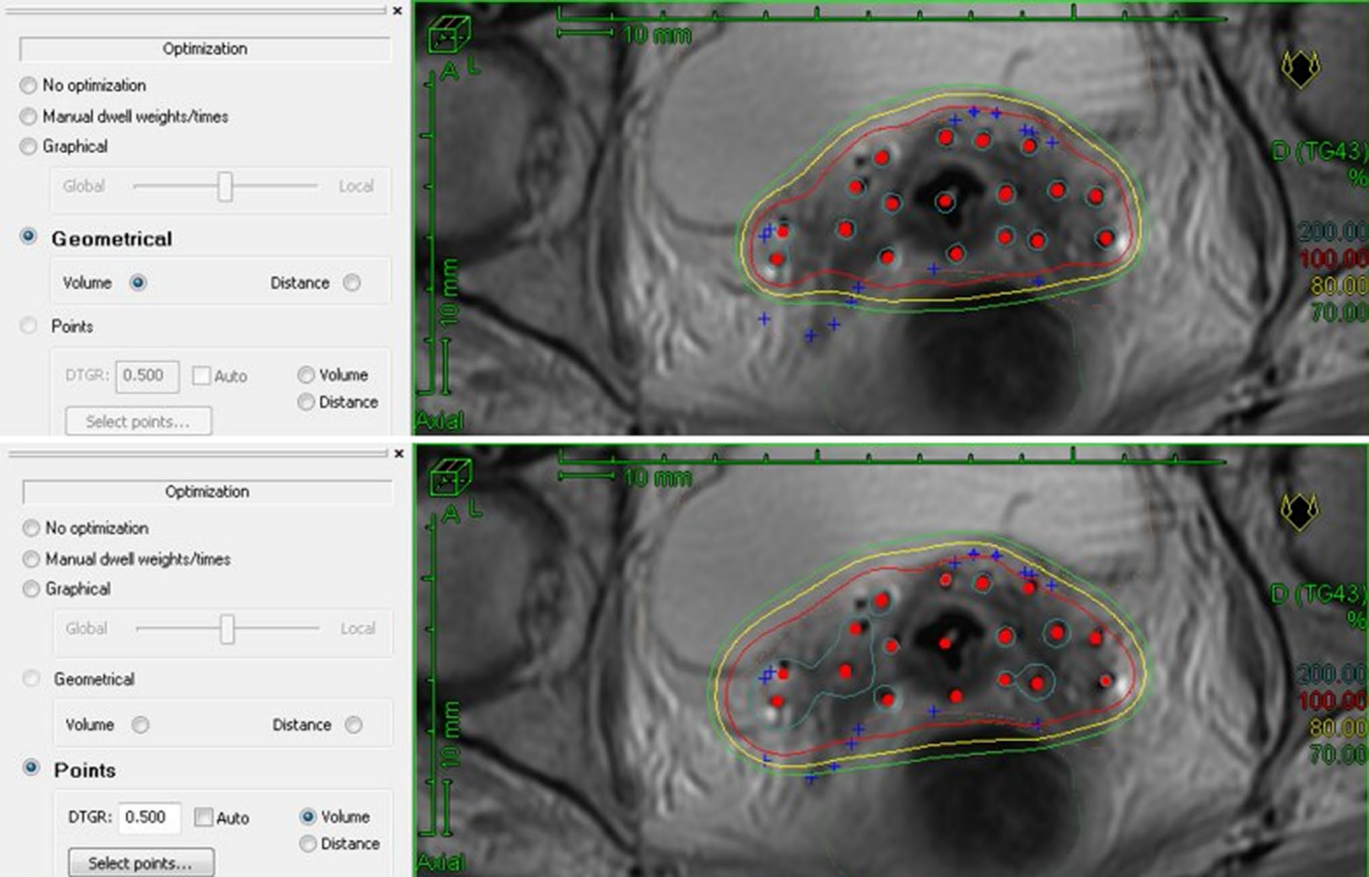
Example showing differences between geometrical and point-based optimisation

A points-based optimisation consists of generating a mesh of points on the surface of the CTV (target points) and then prescribing the nominal dose to these points. However, when using this type of optimisation, it is not uncommon to find hot spots within the CTV. In our experience, the best way to control these hot spots is to use geometric optimisation, which results in small hot spots at the needle boundaries that do not overlap between adjacent needles. Small manual (graphical) adjustments are performed to assure adequate coverage of the CTV (Fig. 6).

#### Interstitial

The implant dwell positions should be selected on the clinical target. Initially, plan optimisation can be performed using automated tools in the TPS, followed by manual optimisation if necessary. Alternatively, manual optimisation can be performed without the need for the automated tools. The dose to 90% of the CTV volume should meet the prescription goals if the CTV is contoured. Otherwise, normal tissue dosimetry should include descriptions of the doses to volumes such as 0.1 cm^3^, 1 cm^3^, and 2 cm^3^ of the bladder, urethra, rectum, sigmoid colon, and small bowel, depending on the location of the lesion. Summation of the EBRT and BT doses can be performed using EQD2 (equivalent dose in 2 Gy fractions) with α/β of 10 Gy for tumour and 3 Gy for normal tissues. During optimisation, the dwell times should be reviewed to ensure that there are no unexpectedly high dwell times. The volume of tissues receiving > 150% of the prescription dose should be limited to the area near the interstitial needles. The use of quality indices to assess conformality and homogeneity is recommended. The conformity index (CI) is defined as (CTVref/VCTV) (CTVref/Vref) where CTVref is the CTV volume receiving a dose equal to or greater than the reference dose, VCTV is the CTV volume, and Vref is the volume receiving a dose equal to or greater than the reference dose. The homogeneity index (HI) is defined as the fraction receiving a dose between 100 and 150% of the reference dose [58].

## Dose (fraction/total dose/frequency)

### Cervix intracavitary/interstitial hybrid HDR-BT

Currently, the most common treatment scheme is the one described in the EMBRACE study: 4 fractions of 7 Gy (two implants of 2 fractions each), administered at weeks 6 and 7 of the treatment. Alternatively, this can be administered weekly, with four implants of one fraction each. As noted in clinical guidelines, other schemes have also been used (e.g., 5 fractions of 6 Gy) [1, 6].

Dose recommendations are based on clinical results of the retroEMBRACE and EMBRACE studies. These two large studies (> 2000 patients) have provided sufficient data to make dose recommendations to the GTV and HR-CTV that correlate with local control.

Numerous reports have described the correlation between OAR doses and treatment-related morbidity. In most Spanish centers, we the dose recommendations, including the recommended limits (minimum doses for the GTV and HR-CTV; maximum for OARs) and the optimal doses from EMBRACE II PROTOCOL (https://www.embracestudy.dk) [69].

For the sigmoid and bowel structures, these dose constraints are valid in case of non-mobile bowel loops resulting in the situation that the most exposed volume is located at a similar part of the organ
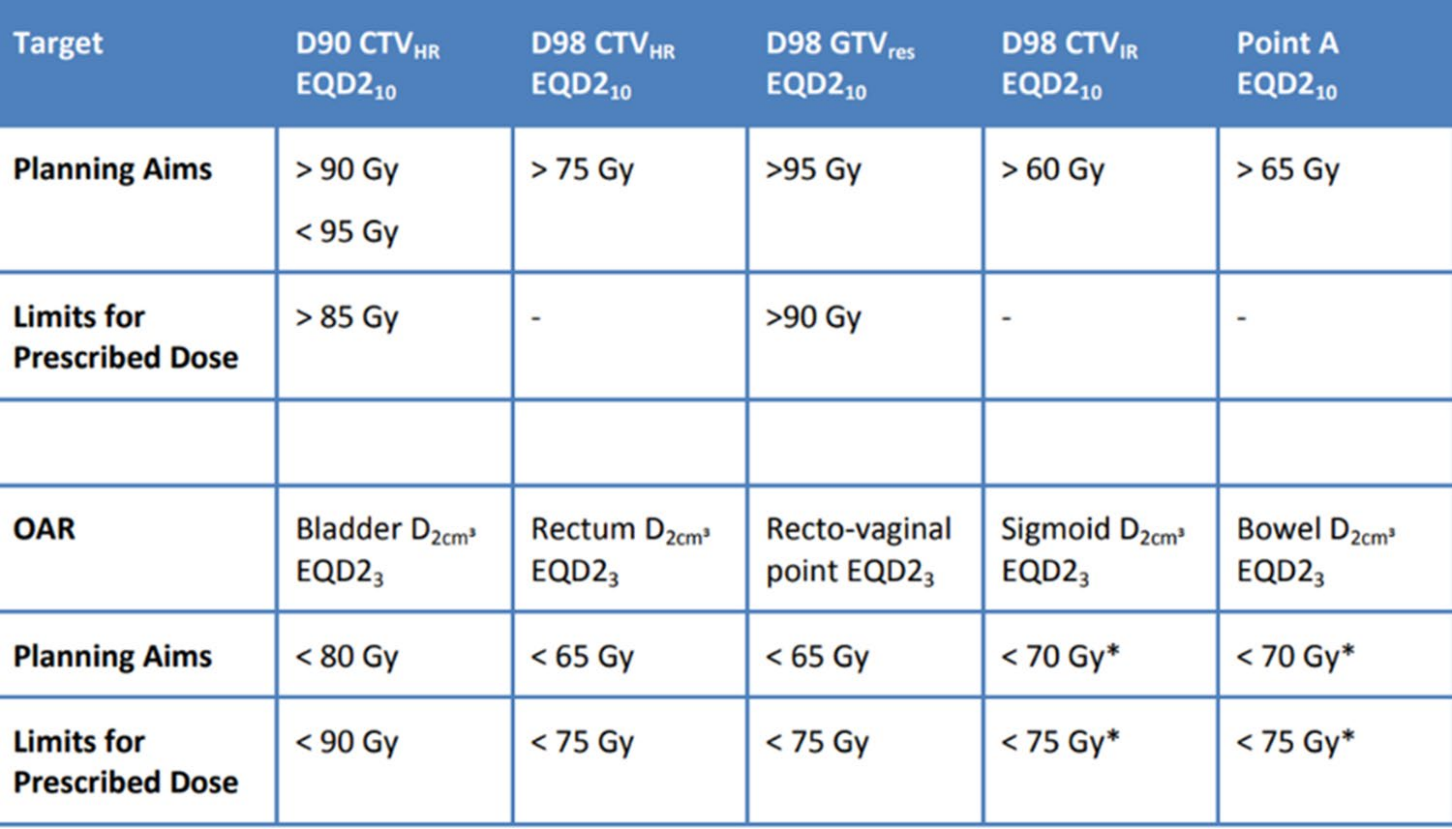


### Cervix perineal templates

Most published recommendations and guidelines—including the those from the ABS—recommend administering five or more fractions [70]. Nonetheless, the dose and fractionation scheme are highly dependent on the treating centre’s experience. The schemes reported to date include the following: 3.5 Gy × 9 fractions (fr); 4.25 Gy × 7 fr; 5 Gy × 5 fr; 3 Gy × 9 fr; 4.5 Gy × 5 fr; and 4 Gy × 6 fr. Given the lack of standardisation, the fractionation schedule must be combined with the EBRT dose using the EQD2 formula.

### Primary vaginal malignancies/vaginal recurrences

Most consensus statements recommend that clinicians to determine the prescription based on their previous experience in cervical and vaginal cancer. Doses should be converted to EQD2 using the linear quadratic model (α/β ratio of 10 Gy for the target, and 3 Gy for OARs). For pulsed dose rate (PDR) BT, a repair half-time of 1.5 h should be applied for both the target and the OARs.

Although precise recommendations for dose and fractionation have not yet been defined, some authors suggest that the combined total dose (EBRT plus BT) to the residual tumour should be > 70 Gy [71]. The latest report of the GYN GEC-ESTRO vaginal cancer task group reported higher local control rates in large tumours when the dose above 80 Gy. In terms of dose/fractionation and number of applications, most centres perform a single application with 2–3 fractions of 6–7 Gy each (median CTV dose: 79 Gy).

In cases with vaginal recurrence in a previously irradiated area, reirradiation may be possible, but the treatment must be individualised. Unfortunately, no recommendations are currently available in this clinical setting, and the only available published data are retrospective. In 2020, the ABS published a literature review on reirradiation in gynaecologic cancers, showing that HDR doses > 40 Gy achieved reasonably good local control and toxicity outcomes. In terms of dose and fractionation, the scheme used in many centres is 4–6 Gy in 5–10 fractions, administered twice daily. To our knowledge, no reports have been published to date on the use of PDR in this setting [72, 73].

### Interstitial vulvar BT

Currently, several different fractionation schemes are available for interstitial vulvar BT. However, all of the current data are based on retrospective studies (3, 51–53). As mentioned above, doses should be reported after conversion into EQD2 of 2 Gy/fraction (α/β 10 Gy for the tumour, half-time of 1.5 h).

When BT is administered as an adjuvant treatment (i.e., boost) after EBRT, we recommend a dose of 18–21 Gy (6–7 fractions of 3 Gy/ twice daily), which is equivalent to an EQD2 Gy: α/β10/3 19.5/21.6–23.6/27.3. A five-fraction schedule of 3.5 Gy twice daily can also be used. If adjuvant ISBT is administered alone, the recommended dose is 40.5 Gy (9 fractions of 4.5 Gy each, twice daily).

When ISBT is used to deliver a boost to the primary vulvar tumour after EBRT, we recommend a dose of 21–24 Gy (7–8 fractions of 3 Gy/twice daily), which is equivalent to EQD2 Gy: α/β10/3 22.7/25.2–26/28.8. Alternatively, a schedule of 6–7 fractions of 3.5 Gy or 5 fractions of 4 Gy (both administered twice daily) can also be used. If BT is delivered as monotherapy, the recommended dose is 45 Gy (10 fractions of 4.5 Gy/ twice daily). The volumes receiving 90% (V90), 100% (V100), 150% (V150), and 200% (V200) of the prescribed dose should be reported.

For postoperative recurrences, the recommendations are the same as in de novo tumours [51, 52, 73]. In recurrences after previous irradiation, it is essential to personalise the treatment approach, especially given the scant published data in this clinical setting [3, 52, 53, 74, 75]. The total dose (EBRT or BT alone, or combined treatment) should be expressed in radiobiologically-equivalent doses of 2 Gy/fraction; always taking into account the 2 Gy biologically-effective dose (BED) received for the previous treatment and the time elapsed between the two treatments.

The most important OAR is the urethra. The recommended approach to evaluating the dose administered dose to this organ is to assess D2cc and D0.1 cc based on the dose-volume histogram derived from the 3D dose distribution. Other OARs that should be considered are the anus and the clitoris. Given the lack of concrete data in the literature, the ALARA criteria (*as low as reasonably achievable*) should be followed.

## Role of The dosimetrist in gynaecologic interstitial brachytherapy

The dosimetrist is involved in many steps of gynaecological BT. Some of the main responsibilities are as follows:

- Quality control of applicators and transfer tubes:
  - Applicators: verify that there is no deformity and verify the distal positions and offset to ensure that their real behaviour is exactly as planned in the reconstruction.
  - Transfer tubes: check that no folds or creases are present, and that the tubes properly connect to the indexer and applicator.
- Patient treatment:
  - Active identification of the patient to ensure an exact match between the planned treatment session and the specific patient.
  - Connect the transfer tubes to the applicators according to the dosimetric indications.
  - For all fractions, check that the applicators are in perfect condition and shape and that the source can pass through the applicators without any risk.
- Create a comfortable and safe climate for the patient before and after the treatment to avoid radiological incidents and no patient movement during treatment delivery.

## Results of the Spanish survey

### Conclusions of the Spanish survey:

1. Of the centres that perform gynaecologic brachytherapy, two-thirds (67%) report using the interstitial technique, although all of the professionals surveyed recognise the importance of the interstitial component.
2. Among the centres that employ an interstitial component in gynaecologic BT (*n* = 24), the most common applications are for the cervix (96%), vagina (87.5%), vulva (75%), and relapses (81%).
3. All of the centres (100%) have HDR. Two centres routinely use PDR.
4. 81.5% of the centres use MRI for gynaecological BT planning, at least for the first fraction in the first implant.
5. In 59.3% of centres, image control is taken before the next fraction for the same implant. Three centres (11.1%) use only one fraction per implant (i.e., a new implant is performed for each fraction).
6. The most common applicator type is the Utrecht (*n* = 18; 75%), but other applicators are also employed, including freehand needles, tubes, and personalised templates (see responses to question 5)
7. Relapses: if the target area has not been previously irradiated (EBRT), all of the centres administer combined radiotherapy (EBRT and BT). For reirradiation, most centres (69.2%) use BT alone
8. Fractionation: Slightly more than half (*n* = 14/24; 58%) of centres use four fractions of 7 Gy, following GEC-ESTRO recommendations. Among the other centres, a wide range of different fractionation schemes are used. In recurrent disease, hen BT is used as monotherapy for reirradiation, the administered dose must be ≥ 40–50 Gy in 9–10 fractions, taking into account the EQD2 (see response to question 8).
9. Planning: 42% of centres use the Manchester system (modified pear), and 54% use inverse planning. For interstitial vulvar implants, 37.5% of centres use the Paris system.

### After an in-depth discussion of the survey results, the working group reached the following consensus-based recommendations:

1. In cervix BT, the interstitial component is important— even when the HR-CTV is small—to improve dose distribution, especially doses to the OARs.
2. Centres that lack experience in treating large cervical tumours or other gynaecologic cancers (vagina, vulva, and/or recurrences) should refer patients to experienced, high volume centres.
3. Ideally, MRI should be performed after completion of EBRT but prior to starting BT to assess residual disease and to perform preplanning ( especially in relapses) for IGBT.
4. During BT implantation, we recommend ultrasound guidance, both transabdominal and transrectal/transvaginal (depending on the clinician’s experience), for image-guided adaptive BT. Fiducial markers are recommended, if appropriate, to mark the borders.
5. Each centre should select the type of applicators they are most familiar with (Utrecht, Ring, etc.), without precluding the use of freehand needles or personalised templates.
6. Before administration of the subsequent BT fraction, imaging (either CT or x-ray) should be performed for control purposes.
7. In vaginal and vulvar tumours, interstitial needles/plastic tubes are recommended, together with perineal templates or freehand needles/tubes, in accordance with GEC-ESTRO recommendations.
8. For the treatment of recurrent disease, if the target/tumour area has not previously received full EBRT doses, a small field (EBRT) should be applied to cover the macroscopic disease with margins. However, if the entire EBRT dose has been applied previously, including the full dose to the OAR, then we recommend administering BT alone.
9. For cervical cancer, we recommend the GEC-ESTRO scheme: 45 Gy of EBRT + BT in 4 fractions of 7 Gy in two implants (to administer ≥ 85 Gy to D90 HR-CTV); if BT monotherapy is used for reirradiation, we recommend ≥ 40–50 Gy in 9–10 fractions (equivalent to EQD2 60 Gy).
10. For cervical cancer planning, we recommend starting with the Manchester system (modified pear) and adding a weight of no more than 20% for the interstitial needles. Detailed recommendations developed by the SEFM are available (Pérez-Calatayud 2018).
11. For inverse planning techniques, the late effects in hot spots have not been well-characterised. Therefore, caution is warranted. For pure interstitial implants, we recommend using the modified Paris System. We also recommend revising the source stopping times to ensure there are no great source steps.

## Conclusions

The interstitial component in gynaecological BT plays an essential role in the treatment of cancers of the cervix, vagina and vulva, both in primary tumours and recurrent disease. Appropriate training is highly recommended.

## Data Availability

I declare that the data, the pictures and all the writing are original and fruit of our experience.
