# Correction to: Recommendations of the Spanish brachytherapy group of the Spanish Society of Radiation Oncology and the Spanish Society of Medical Physics for interstitial high‑dose‑rate brachytherapy for gynaecologic malignancies

**DOI:** 10.1007/s12094-022-03051-y

**Published:** 2023-01-02

**Authors:** Cristina Gutiérrez Miguélez, Silvia Rodríguez Villalba, Elena Villafranca Iturre, Naiara Fuentemilla Urio, Jose Richart Sancho, Sofía Córdoba Lago, Francisco Pino Sorroche, Ruth Gracia Lucio, Antonio Herreros Martínez, Dina Najjari-Jamal, GEB (Spanish Brachytherapy Group)

**Affiliations:** 1 Radiation Oncology Department, Institut Català d’Oncologia, Facultat de Medicina i Ciències de la Salut, Universitat de Barcelona (UB) Catalonia, Hospitalet de Llobregat, Spain; 2 Radiation Oncology Department, Hospital Clínica Benidorm, Benidorm, Spain; 3 Radiation Oncology Department, Complejo Hospitalario de Navarra, Pamplona, Spain; 4 Radiation Oncology Department, Hospital Universitario San Juan, Alicante, Spain; 5 Radiation Oncology Department, Hospital Universitario Puerta de Hierro, Madrid, Spain; 6 Radiophysics Department, Institut Català d’Oncologia, Hospitalet de Llobregat, Catalonia Spain; 7 Radiophysics Department, Hospital Clínic de Barcelona, Barcelona, Catalonia Spain

**Correction to: Clinical and Translational Oncology** 10.1007/s12094-022-03016-1

In this article the author names Silvia Rodríguez Villalba, Elena Villafranca Iturre and Ruth Gracia Lucio were incorrectly written as Rodríguez Rodriguez Villalba, Villafranca Villafranca Iturre and Garcia Gracia Lucio.

The original article has been corrected.

